# The shape of abundance distributions across temperature gradients in reef fishes

**DOI:** 10.1111/ele.13222

**Published:** 2019-02-10

**Authors:** Conor Waldock, Rick D. Stuart‐Smith, Graham J. Edgar, Tomas J. Bird, Amanda E. Bates

**Affiliations:** ^1^ Ocean and Earth Sciences National Oceanography Centre Southampton University of Southampton Waterfront Campus Southampton SO14 3ZH UK; ^2^ Department of Life Sciences Natural History Museum London UK; ^3^ Institute for Marine and Antarctic Studies University of Tasmania Hobart TAS 7001 Australia; ^4^ Geography and Environment University of Southampton Southampton SO17 1BJ UK; ^5^ Department of Ocean Sciences Memorial University of Newfoundland St John's NL Canada

**Keywords:** Abundant‐centre hypothesis, ecological performance, geographic range, niche partitioning, realised niche, species distribution, species distribution model, thermal performance curve, thermal‐abundance distribution

## Abstract

Improving predictions of ecological responses to climate change requires understanding how local abundance relates to temperature gradients, yet many factors influence local abundance in wild populations. We evaluated the shape of thermal‐abundance distributions using 98 422 abundance estimates of 702 reef fish species worldwide. We found that curved ceilings in local abundance related to sea temperatures for most species, where local abundance declined from realised thermal ‘optima’ towards warmer and cooler environments. Although generally supporting the abundant‐centre hypothesis, many species also displayed asymmetrical thermal‐abundance distributions. For many tropical species, abundances did not decline at warm distribution edges due to an unavailability of warmer environments at the equator. Habitat transitions from coral to macroalgal dominance in subtropical zones also influenced abundance distribution shapes. By quantifying the factors constraining species’ abundance, we provide an important empirical basis for improving predictions of community re‐structuring in a warmer world.

## Introduction

Amongst the most fundamental questions in ecology is how an organism's performance is affected by gradients in environmental conditions. Ecological performance (e.g. fitness, demographic rates, abundance, occupancy) in geographic space is often difficult to attribute to environmental variation because many entangled processes act at once (Gaston 2009; Pironon *et al*. 2016). Given the importance of temperature in structuring life across biological scales, from biochemical reactions to organism behaviour and species’ interactions (Dell *et al*. 2011), characterising the role of temperature in driving realised ecological performance is essential for predicting species’ responses to warming and environmental variability (Deutsch *et al*. 2008).

Species’ abundance is expected to be greatest at the centre of their environmental niche if performance declines outside of particular ‘optimal’ environmental conditions (Brown *et al*. 1995; Pironon *et al*. 2016). Many explanations exist for this ‘abundant‐centre’ effect, with mechanisms operating from small to large scales (e.g. physiology, environmental auto‐correlation, Brown 1984; Pironon *et al*. 2016). However, assumptions underlying abundant‐centre effects have been questioned for decades, and can be violated due to various ecological and evolutionary factors including: (1) fine‐scale environmental heterogeneity, (2) local adaptation, (3) physical barriers to dispersal truncating geographic ranges, (4) geographic availability of niche space, (5) habitat gradients and (6) species’ interactions (Sagarin *et al*. 2006). Therefore, the distribution of abundance across environmental gradients is often complex, and abundance patterns have frequently been inconsistent with the abundant‐centre hypothesis (Sagarin & Gaines 2002b; Pironon *et al*. 2016; Dallas *et al*. 2017; Santini *et al*. 2018).

Moreover, it may be unrealistic to assume that species from tropical and temperate systems – which experience markedly different temperature regimes – will display similar abundance structure across thermal gradients. For example, abundance may peak closer to warm thermal distribution edges when temperature variation is low, such as the tropics. Tropical species investigated generally have narrow thermal safety margins – they live nearer their thermal upper limits for physiological and demographic rates (Angilletta *et al*. 2010; Morley *et al*. 2012). In contrast, temperate species experience higher seasonal and short‐term temperature variation and may have optimal temperatures below upper limits.

Compelling ecological and physiological hypotheses predict the shape of abundance across thermal distributions, but defining the peaks and edges of thermal distributions presents practical challenges. Both require high‐resolution survey data across large spatial scales (Bates *et al*. 2014; Knouft 2018). Abundance data are highly variable and strongly affected by sampling errors and biases. Sites of similar temperatures frequently differ in many other factors affecting local abundance. Thus, the influence of temperature may not be obvious when examining mean local abundance. Instead, a signal may be more easily detected from upper abundance limits, given enough data (Cade & Noon 2003; Vanderwal *et al*. 2009), and more compelling tests of abundant‐centre effects have been described by modelling maximum abundance (Langlois *et al*. 2012; Knouft & Anthony 2016; Martinez‐Gutierrez *et al*. 2018).

Here, we use the Reef Life Survey (RLS) data – a standardised, well replicated and globally distributed species‐level census of whole shallow reef fish communities – to overcome data consistency and sample size issues which have been prohibitive in previous analyses. First, we empirically quantify the variation in abundance across 702 species’ thermal distribution using multiple approaches – we call this the ‘thermal‐abundance distribution’. Second, we test for systematic differences in the shape of thermal‐abundance distributions in fishes from tropical vs. temperate guilds, accounting for limitations due to habitat availability. Overall, we find abundance is consistently related to temperature, with peaks in performance indicating existence of ‘thermal optima’ for ecological performance. The position of species’ peaks along temperature gradients varies, however, resulting in skewed thermal‐abundance distributions, with a majority of species that are most successful between the centre to warm‐range edges.

## Material and Methods

We evaluated the thermal‐abundance distribution for individual reef fish species and quantified how many species showed abundant‐centre patterns vs. asymmetrical or ‘no‐trend’ shapes (*Categorical assessment of thermal‐abundance distribution shape*). We then standardised abundance and temperature across all species’ geographic ranges and analysed the mean shape of thermal‐abundance distributions (*Quantifying the average shape of thermal‐abundance distributions*). Finally, we modelled variation between tropical and temperate guilds in the shape of species‐specific thermal‐abundance distributions (*Quantifying structure in the thermal‐abundance distribution shape*). See Fig. S1 for a schematic of all analyses.

### Data sources

**Figure 1:**
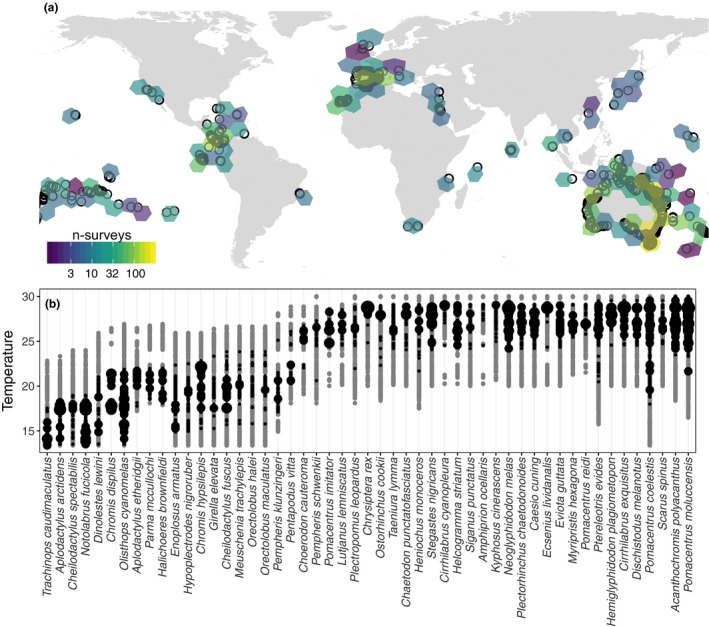
Distribution of Reef Life Survey (RLS) sites across geographic and thermal gradients. (a) Geographic distribution and intensity of RLS sampling used in these analyses; sites across the globe are aggregated to equal area hexagons (*n* = 3132 sites). Points show distribution of RLS sites. (b) Sampled thermal distributions (grey points) relative to occurrences (black points) for 50 example species, sampling often extends beyond range edges.

The abundances of all fishes present along transects were counted by trained RLS participants between 2007 and 2016 (Fig. 1) following strict data quality control (Edgar & Stuart‐Smith 2014, http://www.reeflifesurvey.com). For each species, the abundance for non‐cryptic/adult individuals (> 40% maximum body length, Froese & Pauly 2000) was summed within individual transects, and then averaged among transects at each RLS ‘site’ (minimum 200 m apart) providing site‐level mean densities per 500 m^2^. Species with < 30 abundance records or an observed thermal range of <3°C (mean sea surface temperature; see below) were deemed to have inadequate data for the modelling approaches used here. This gave us 98 422 abundance estimates for 702 fish species at 3120 sites. We defined species’ absences from a circular buffer with a radius of 10° latitude/longitude around each RLS site, recording zero abundance at sites surveyed at which (1) the species was not observed, but (2) was observed elsewhere within this buffer (Bivand & Rundel 2018). While lack of observed presence may not be a ‘true absence’, they represent locations at which a given species was in insufficient local abundance to have been detected in the standardised surveys. This resulted in a total of 781 983 observations for analysis.

We matched the location of each site with sea surface temperature (SST) values at a ~ 5 km^2^ scale (NOAA Coral Reef Watch, 2018, see Figs S2 and S3 for comparison to additional SST resources and metrics). We calculated 2‐year mean annual‐site temperature from daily SST records. This period covered the influence of temperature on individual survival and recruitment processes over multiple generations, which in turn influences population size. Covariate data were obtained from the Marine Socio‐Ecological Covariate dataset (Yeager *et al*. 2017) and Bio‐ORACLE v2.0 (Assis *et al*. 2018; see Table S1). In addition, we estimated species‐specific habitat associations using the total % cover of macroalgae or live coral from 20 photo quadrats from the same transects surveyed for fishes. We estimated mean transect depth from multiple surveys to obtain a site average depth. Finally, thermal guilds were defined from the peak of modelled maximum abundance (*T*_*opt*_, see *Categorical assessment of thermal‐abundance distribution shape*). Species were considered ‘temperate’ when *T*_*opt*_ < 23°C and ‘tropical’ when *T*_*opt*_ > 23°C based on the naturally occurring thermal guild separation previously observed by Stuart‐Smith *et al*. (2015, 2017).

For all species, we estimated semi‐quantitative scores for confidence in values of *T*_*opt*_, and the minimum and maximum temperatures at range limits (*T*_*min*_ and *T*_*max*_). Methods for confidence scores are provided in online supporting materials (Appendix S1) and the derivation of *T*_*opt*_, *T*_*min*_ and *T*_*max*_ is described in sections *Categorical assessment of thermal‐abundance distribution shape* and *Quantifying structure in the thermal‐abundance distribution shape*. Sensitivity analyses with only ‘high‐confidence’ species (*n = *181 species) supported our main results.

### Categorical assessment of thermal‐abundance distribution shape

We used a two‐stage residual analysis to model abundance variation across each species range to handle the effects of multiple covariates on abundance without risk of model overfitting – thereby also retaining a focus on the effects of temperature. We first accounted for the influence of covariates, other than temperature, using generalised linear models fitted with a zero‐inflated Poisson (ZIP) error structure. For each species independently, we ran species‐level principal component analyses (PCA) including factors related to water chemistry (e.g. O_2_, phosphate, nitrate), oceanography and bathymetry (e.g. current velocity), ecology (e.g. productivity, reef area) and human pressures (e.g. human population density) amongst others (see Table S1). We used PCA because the goal of our first‐stage analysis was to account for as many of the factors as possible that potentially affected each species’ local abundance before we tested the effect of temperature – we did not test for the specific effect of each covariate. PC1 was related to site temperature, and therefore it is important to first account for these sources of covariation to identify the independent influence of temperature on abundance. To avoid model overfitting for each species, we only included PCA axes explaining > 10% environmental variation experienced by species across their range. In addition to these PCA axes derived independently for each species, we also included several other covariates in our first‐stage models: site depth, protection status scores (Edgar *et al*. 2014) and sampling intensity (calculated as the number of survey sites sampled per degree temperature across a species' geographic range). Across all RLS sites, the most important sources of environmental variation were human population density, reef area, dissolved‐O_2_ and productivity (see Fig. S4).

**Figure 2:**
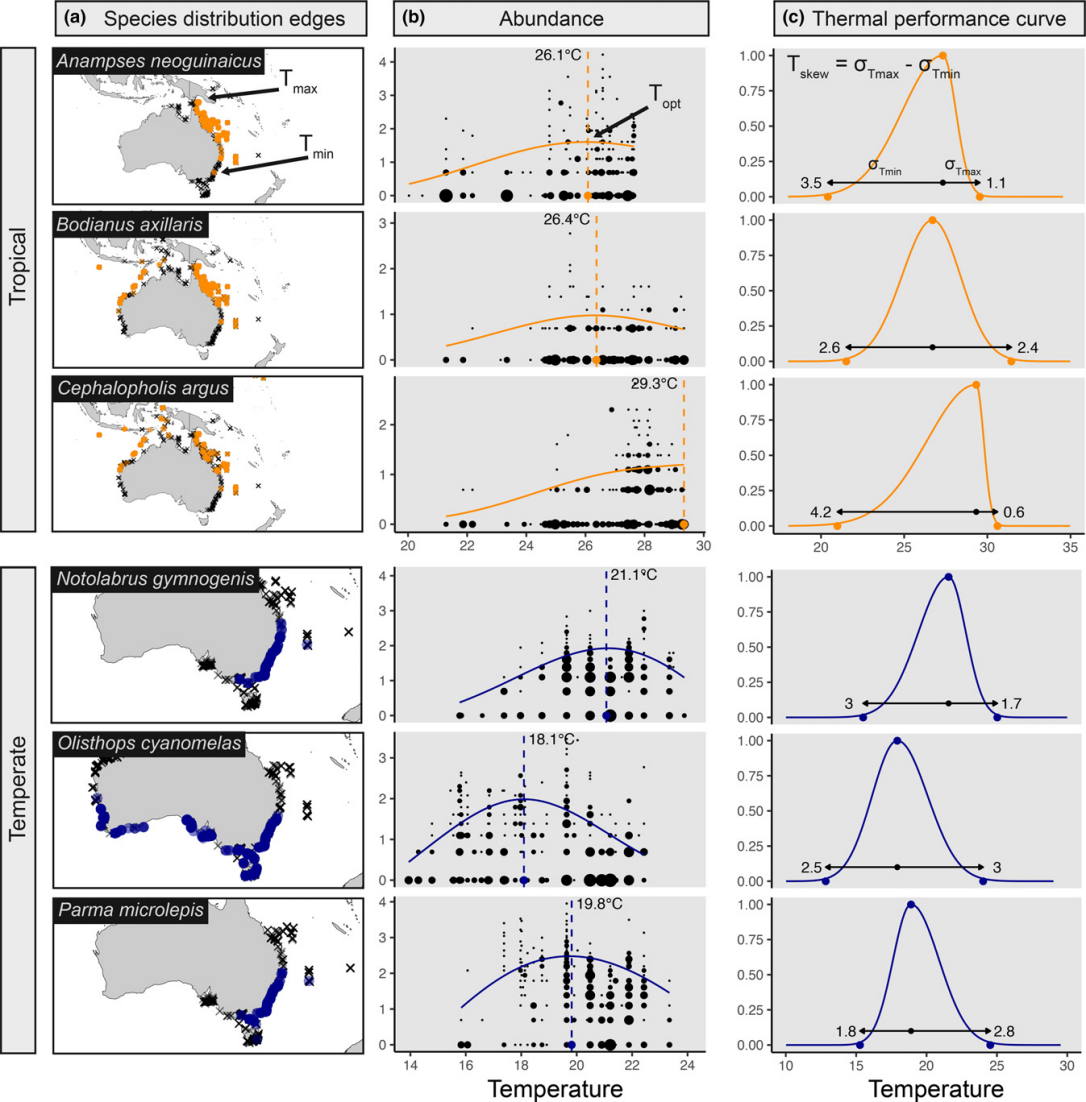
Overview of modelling tools and parameters used to characterise the shape of species' temperature‐abundance distribution, including example models from the species‐specific analysis in tropical (orange) and temperate (blue) guilds. (a) *T*_*min*_ and *T*_*max*_, that is, species distribution edges, were derived from species’ geographic distributions. (b) Illustrative models conceptualising estimation of *T*_*opt*_ which was derived from quantile generalised additive models (qgam) fit to log10 species abundance (note that in main analysis qgam is performed on species' residual‐abundance in a 2‐stage analysis). Point sizes equate to the number of overlapping points. (c) We use these parameters to define a *T*_*skew*_ for 702 species represented by a split‐Gaussian function here (see methods and eqn. 1 for full details).

For each species we extracted the residuals from the first‐stage models, and then modelled the relationship between temperature and residual‐abundance using quantile generalised additive models in the R package ‘qgam’ (Fasiolo *et al*. 2017). The use of generalised additive models rather than linear models allowed a flexible fit to highly variable abundance data. We fitted temperature as a smooth term at the 80th quantile of residual‐abundance, thus we modelled maximum residual‐abundance without needing to estimate maximum abundance within a temperature ‘bin’. We used *k = *4 degrees of freedom in our regression spline so models were robust to outliers and fitted curves were constrained, to some extent, in non‐linearity. We also limited the number of absences in each species to equal the number of abundance records. Absences are far more frequent and could overwhelm the shape of abundance distributions, the number of absences also varied by orders of magnitude between species so our approach balanced the number of presences vs. absences for each species. When absences were constrained, we bootstrapped predictions by re‐running models to random absence subsets 25 times to avoid spurious estimates of T_opt_ that depended on which absences were excluded. We defined the *T*_*opt*_ of each species as the temperature of peak abundance (Fig. 2). In a test of robustness, *T*_*opt*_ values derived from models including and excluding covariates (i.e. a one‐stage analysis) were highly correlated with a slope of 1.03 ± 0.01 (*r* ^2 ^= 0.90, Fig. S5). For each species, we tested for the presence of spatial auto‐correlation in model residuals by comparing correlations between site pairwise distances and residual Euclidean distances using Mantel tests with 999 permutations. Correlations between these two distance matrices were, on average, very weak (0.08 ± 0.08). Thus, type‐1 errors are unlikely to be inflated due to underestimated number of degrees of freedom. Including a spatial auto‐correlation term when covariates are highly auto‐correlate can lead to a focus on local factors driving abundance, here we retain a focus on large scale covariates (i.e., temperature) by not including a spatial auto‐correlation term in our final models (Diniz‐Filho *et al*. 2003).

From these models we defined four thermal‐abundance distribution shapes, measured by the drop of abundance at the edges of thermal distributions (Sagarin & Gaines 2002a):

1. *No‐trend*: neither thermal distribution edge falls to < 75% of maximum modelled abundance,
2. *Abundant‐centre*: both thermal distribution edges fall to < 75% of maximum modelled abundance,
3. *Warm‐skewed*: only warm thermal distribution edge does not fall to < 75% of maximum modelled abundance,
4. *Cool‐skewed*: only cool thermal distribution edge does not fall to < 75% of maximum modelled abundance,

We assessed differences in the proportion of species in each group using a chi‐squared goodness‐of‐fit test, and tested whether a threshold of 50% maximum abundance influenced our results.

### Quantifying the average shape of thermal‐abundance distributions

To quantify the overall shape of thermal‐abundance distributions in tropical and temperate guilds, we aggregated all species into a single thermal‐abundance distribution model. Specifically, this analysis tested whether the decline above and below *T*_*opt*_ occurred at comparable rates, even if species thermal distributions were truncated by biogeographic factors. We standardised both *x*‐ and *y*‐axes (temperature and abundance respectively) in order to allow comparison of curve shape among species with different absolute values of abundance and different thermal distribution widths. We standardised the width of the thermal distributions among species by scaling temperatures to the mean of $\sigma_{T_{\min}}$ and $\sigma_{T_{\max}}$ (defined in *Quantifying structure in the thermal‐abundance distribution shape*). We then centred this distribution by *T*_*opt*_ to produce a distribution of temperatures centred on 0 (*T*_*opt*_ **= 0). We standardised the range of local abundances by the maximum abundance across a species geographic range to constrain the absolute height of the *T*_*opt*_ peak between 0 and 1. We only used abundance records (i.e. excluded absences). Within each 0.1 temperature bin we estimated, for each species, the 99th percentile of standardised abundance and fitted our model to these maximum abundance values.

Next, we modelled temperature‐related ecological performance, Performance (*T*), directly from the relationship between abundance and temperature (transformed as described above) across all species, using the following split‐Gaussian function:

(1) $$\mathrm{Performance}\left(T\right)=\left\{\begin{array}{c} c\times e^{-{\left(\frac{T-T_{\mathrm{opt}}}{\sigma_{T_{\min}}}\right)}^{2}}T<T_{opt,}\\ c\times e^{-{\left(\frac{T-T_{\mathrm{opt}}}{\sigma_{T_{\max}}}\right)}^{2}}T>T_{\mathrm{opt}} \end{array}\right.$$

where *T* is the temperature and *c* is the scaling parameter which defines the height of the abundance peak at *T*_*opt*_. This split‐Gaussian function modelled abundance as a function of temperature using separate Gaussian functions above and below *T*_*opt*_, the temperature of peak abundance. The rate of change in abundance across thermal distributions is described by separate standard deviations above ($\sigma_{T_{\max}}$) and below ($\sigma_{T_{\min}}$) *T*_*opt*_. The thermal‐abundance distribution shape parameter *T*_*skew*_ was estimated as $\sigma_{T_{\max}}-\sigma_{T_{\min}}$ from the above equation.

We estimated *c, T*_*opt*_, $\sigma_{T_{\min}},\sigma_{T_{\max}}$ and *T*_*skew*_ using MCMC sampling (prior values are provided in Table S2), and fitted models using JAGS (to provide a flexible framework to define this split‐Gaussian functional form) with the package ‘r2jags’ (Su & Yajima 2012). We fitted models with four chains of 10 000 iterations each, a burn‐in of 2500 iterations and a thinning of 5. We visually assessed mixing and stability of MCMC chains for all parameters, as well as confirming that the Gelman‐Rubin convergence diagnostic statistic was < 1.01 to indicate that models were fully converged. Statistical significance was inferred from assessing the 95% credible interval of parameter posterior distributions. We fitted this model separately to temperate and tropical guilds to obtain simple approximations of thermal‐abundance distribution shapes.

### Quantifying structure in the thermal‐abundance distribution shape

In addition to the qualitative assessment of thermal‐abundance distribution shape (i.e. *Categorical assessment of thermal‐abundance distribution shape*) and an average thermal‐abundance distribution shape for each thermal guild (i.e. *Quantifying the average shape of thermal‐abundance distributions*), for each species we estimated a quantitative continuous parameter of thermal‐abundance distribution shape (*T*_*skew*_). This was based on the distance of thermal optima (*T*_*opt*_ defined in *Categorical assessment of thermal‐abundance distribution shape*) to thermal distribution edges (*T*_*min*_ and *T*_*max*_ – defined below). We note this approach assumed species thermal performance followed the shape presented in eqn. 1, an assumption generally well supported in our data (see Results), as well as physiological (Angilletta 2009; Dell *et al*. 2011) and ecological (Boucher‐Lalonde *et al*. 2014) models. Here, we assumed the parameters of eqn. 1 can be derived from abundance (*T*_*opt*_ in *Categorical assessment of thermal‐abundance distribution shape*) and thermal range edges ($\sigma_{T_{\min}}$ and $\sigma_{T_{\max}}$ defined below) that describe the shape of species’ thermal‐abundance distributions. The parameters $\sigma_{T_{\min}}$ and $\sigma_{T_{\max}}$ were derived from *T*_*min*_ and *T*_*max*_, that is, the thermal distribution edges defined from species’ distributions as described below. We set the scaling parameter *c* to 1.

#### Deriving *T*_min_ and *T*_max_

*T*_*min*_ and *T*_*max*_ were estimated from the observed 2.5th and 97.5th quantiles of species thermal distributions for each species (as in Stuart‐Smith *et al*. 2017). We also accounted for the influence of seasonality by defining *T*_*min*_ and *T*_*max*_ as the 2.5th and 97.5th quantiles of minimum and maximum temperatures across species distributions during a 2‐year period. Furthermore, we accounted for the influence of additional covariates on *T*_*min*_ and *T*_*max*_ (and extended species’ geographic distributions beyond sampled sites) by fitting an ensemble of species’ distribution models (SDMs) for each species (details in Appendix S1). We estimated the 2.5th and 97.5th quantiles of species’ predicted thermal distributions from these models – however, our choice of methods to derive *T*_*min*_ and *T*_*max*_ had no qualitative influence on our results (results in Appendix S2). Here, we presented the results for *T*_*min*_ and *T*_*max*_ derived from sampling limits only, excluding the influence of seasonality (Stuart‐Smith *et al*. 2017). We assumed *T*_*min*_ and *T*_*max*_ are the 95^th^ percentiles of a normal distribution with a mean of *T*_*opt*_, and that the *z*‐score of this distribution was 1.96. We then defined the thermal distribution parameters $\sigma_{T_{\min}}$ and $\sigma_{T_{\max}}$, introduced in eqn. 1, as: $\sigma_{T_{\min}}=\left(T_{\min}-T_{\mathrm{opt}}\right)/1.96,\;\text{and similarly for}\;\sigma_{T_{\max}}$.

#### Modelling thermal‐abundance distribution skew

*T*_*skew*_ ($\sigma_{T_{\max}}-\sigma_{T_{\min}}$) was quantified as the imbalance of cool and warm thermal distributions edges from *T*_*opt*_. We follow the terminology of the section *Categorical assessment of thermal‐abundance distribution shape*. That is, where species had *T*_*opt*_ closer to warm thermal distribution edges (*T*_*max*_) we called this ‘warm‐skewed’ and *T*_*skew*_ was negative. Where species had *T*_*opt*_ closer to cool thermal distribution edges (*T*_*min*_) we called this ‘cool‐skewed’ and *T*_*skew*_ was positive. Where species had *T*_*opt*_ in the exact centre between *T*_*min*_ and *T*_*max*_ the skew value was 0.

We estimated the slope of *T*_*skew*_ vs. *T*_*opt*_ within guilds, using linear mixed‐effects models fitted in R using ‘lme4’ (Bates *et al*. 2015; version 1.1–17). We fitted separate models for temperate and tropical guilds. We included *T*_*opt*_ as a linear independent variable. Species’ coral and macroalgae associations were also modelled as independent variables to account for influences of habitat preferences and the geographic patterns in habitat availability on *T*_*skew*_ (Figs S8 and S10). We fitted these covariates as simple additive effects with no interactions. We included taxonomic structure as a nested random intercept of Order, Family and Genus in all models to help account for similarities in traits due to shared evolutionary histories within taxonomic groups. We tested for the influence of these terms by comparing AICc values between models. We also used backwards‐stepwise model selection, comparing between model fits using likelihood‐ratio tests. In addition to the above models, across all species we tested for the potential of non‐linear interactive effect of *T*_*opt*_ with habitat association on *T*_*skew*_ using generalised additive models, fitting this term using tensor product smooths with the R package ‘mgcv’ (Wood 2011) and comparing model fits using AICc values. This modelling approach allowed *T*_*skew*_ to be modelled with a non‐linear interaction between simultaneous gradients in *T*_*opt*_ and species’ habitat associations.

Code and data for all analyses are available online (code available at https://github.com/cwaldock1/RLS-ThermalNiche, data available at https://doi.org/10.6084/m9.figshare.7218104), all analysis were run using the statistical software ‘R’ version 3.4.0 (R Core Team, 2017).

## Results

### Categorical assessment of thermal‐abundance distribution shape

**Figure 3:**
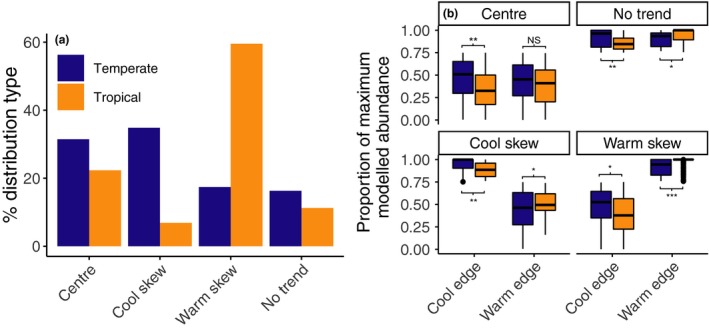
(a) Percentage of each distribution type and (b) associated decline in relative abundance at thermal distribution edges relative to *T*_*opt*_ across thermal guilds. Panels in (b) are separated by thermal distribution types and *x*‐axis shows thermal distribution edges.

Temperature and maximum abundance were significantly related for 75% of the 702 species included in our modelling. The deviance in maximum abundance explained by temperature ranged between 14 and 63%. Thermal‐abundance distributions showed abundant‐centre patterns for 25% of species (Fig. 3), and on average, abundance declined by two‐thirds of maximum abundance at these species’ thermal range edges. Of the remaining species not fitting our ‘abundant‐centre’ criteria, warm‐skewed shapes were common (49%), with fewer cool‐skewed (14%) and no‐trend (13%) relationships (Fig. 3; *X* ^2^ = 237, d.f. = 3, *P* < 0.001).

Abundant‐centre trends were more apparent in widespread species with richest data. Among the ‘high‐confidence’ set of species, there was a more even balance of species having warm‐skewed (48%) and abundant‐centre (38%) thermal‐abundance distribution shapes, with the remaining species displaying cool‐skewed (10%) or no‐trend (3%) shapes (*X* ^2^ = 100, d.f. = 3, *P *< 0.001). Where species are not limited by geographic boundaries at cool‐range limits (i.e. continental margins for southern hemisphere temperate zones) or ‘niche availability’ limits at warm‐range edges (i.e. warmest temperatures in oceans), 97% of species display peak maximum abundances away from the edges of species’ thermal distributions (i.e. *T*_*opt*_ does not = *T*_*min*_ or *T*_*max*_).

We also detected a difference between thermal‐abundance distribution in tropical vs. temperate guilds. The thermal distributions of tropical species were mostly warm‐skewed, whereas temperate species were mostly cool‐skewed or abundant‐centre (Fig. 3). At cool‐range edges, tropical species generally had lower relative abundance than temperate species, but at warm‐range edges temperate species had lower relative abundances than tropical species (Fig. 3b).

### Quantifying the average shape of thermal‐abundance distributions

**Figure 4:**
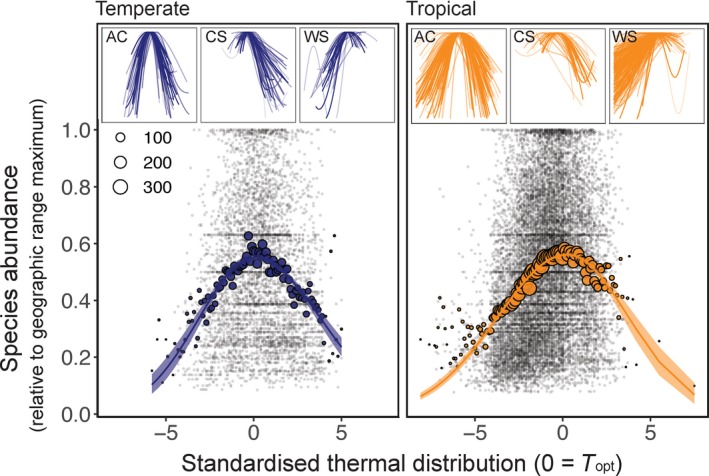
Species abundance across thermal distributions for 702 reef fishes on common scales of abundance and temperature. Abundance declines with temperature deviation from *T*_*opt*_, that is, towards thermal distribution edges. The *x*‐axis represents the number of standard deviations from *T*_*opt*_ (note the scale on *x* is standardised within each species range so is not comparable to an absolute temperature value, see Methods for details). Abundance on the *y*‐axis is the local site abundance as proportion of each species’ maximum abundance across species’ geographic ranges. Small points are individual species’ 99th quantile of relative abundance, and large points are mean values across species within a temperature bin. Main panels show Bayesian model fits, for a split‐Gaussian distribution, and 95% credible intervals. Panel insets show quantile generalised additive model fits for all 702 species. The split‐Gaussian distribution is formed of species' displaying generally abundance‐centre (AC) patterns with a relatively even number of cool skew (CS) and warm‐skew (WS) species forming the net Gaussian shape that has similar rates of change above and below *T*_*opt*_.

Ecological performance displayed a peak at species’ *T*_*opt*_ in both tropical and temperate thermal guilds (Fig. 4). However, ecological performance varied among species within a given temperature bin, and a low proportion of variation was explained by a simple split‐Gaussian model with temperature as a single covariate (*R* ^2^ = 0.07–0.09). When modelled as a species aggregated mean ecological performance within temperature bins, a much higher proportion of variation in ecological performance across species was explained by this very simple model (*R* ^2^ = 0.73–0.75). The shape of this relationship was modelled with high confidence as indicated from narrow credible intervals for parameter estimates (Fig. 4). The overall abundant‐centre pattern across all species is underpinned by those species’ which display an abundant‐centre pattern, combined with species that have warm‐ and cool‐skewed distributions that decline at both distribution edges (but at a similar rate, on average, to the decline of both range edges in abundant‐centre species, see inset in Fig. 4).

The skew in ecological performance was significantly different between guilds. Species in the tropical guild were significantly negatively skewed (*T*_*skew *_= −0.65 ± 0.49), such that *T*_*opt*_ is closer to warm thermal distribution edges. The opposite is true for species in the temperate guild (*T*_*skew *_= 0.88 ± 0.54).

Species are infrequently observed near their maximum observed abundance, even at ‘optimal’ temperatures, as shown by the restricted height of the thermal performance curves at *T*_*opt*_. Species’ abundance at *T*_*opt*_ was only 55–56% maximum abundance observed across a species’ range (Fig. 4).

### Quantifying structure in the thermal‐abundance distribution shape

**Figure 5:**
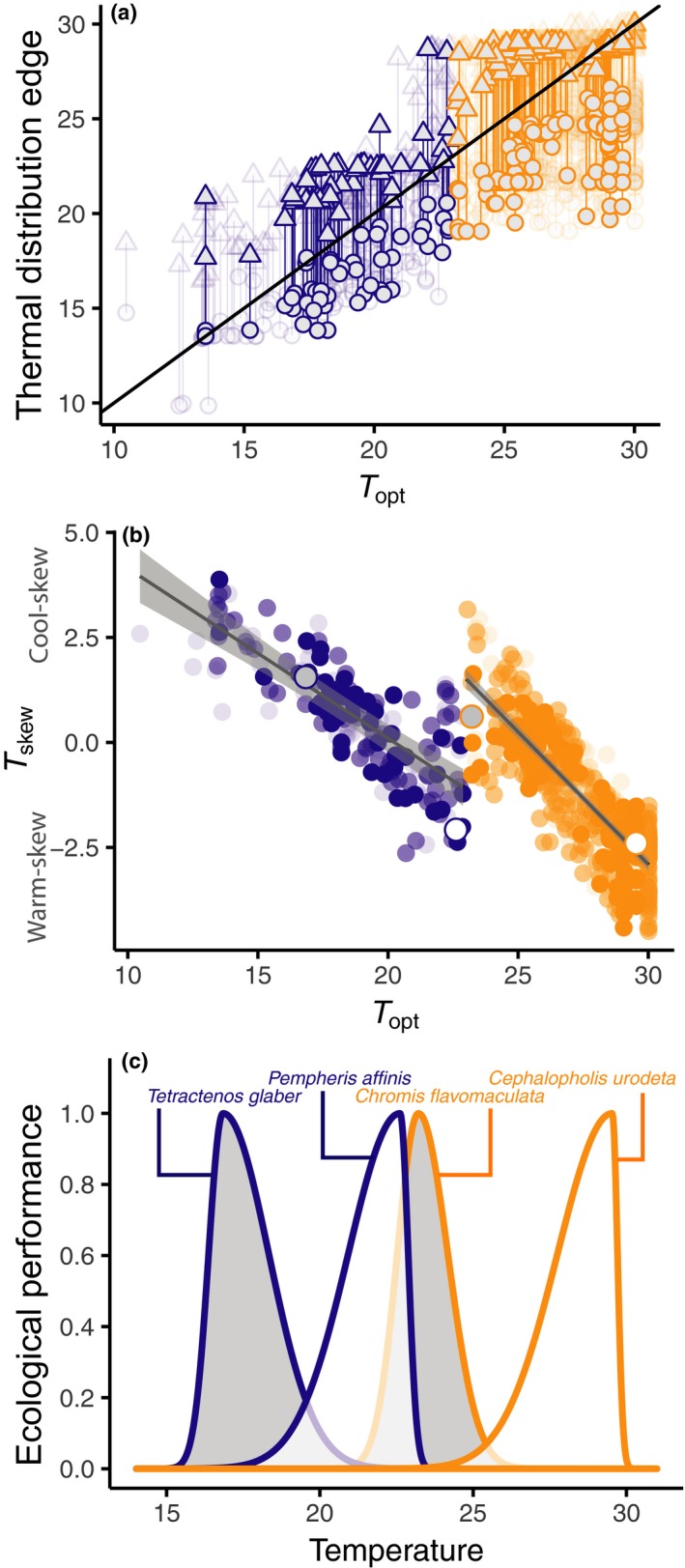
Thermal guilds structure the shape of species’ thermal distributions (blue = temperate, orange = tropical). (a) The placement of thermal niche edges (*T*_*min*_, *T*_*max*_) relative to thermal optima (*T*_*opt*_) which together define realised thermal niche shape (*T*_*skew*_). *T*_*max*_ and *T*_*min*_ show relative invariance across *T*_*opt*_ within each thermal guild but *T*_*opt*_ does vary between species in each guild. Triangles represent upper thermal distribution edges, circles represent lower thermal distribution edges. (b) Shows the negative relationship between *T*_*opt*_ and *T*_*skew*_ which is a consequence of the invariance of thermal distribution edges in comparison with variable *T*_*opt*_ shown in (a). Coloured points in (b) represent partial residuals of species parameter values, excluding the effects of phylogeny and habitat association (coral and macroalgae). Fitted lines are the predicted relationships from a generalised linear mixed‐effects models with associated 95% confidence intervals (see Table S4). Shading indicates confidence scores for species. Large grey or white points represent the species shown as examples in (c). (c) Extreme thermal distribution shapes defined by split‐Gaussian functions, and their associated skew, for temperate and tropical guilds at thermal guild edges. Whilst some species within each guild can have high ecological performance at the thermal guild ‘barrier’ (~ 23°C) in both guilds, some species segregate strongly at this barrier and do not occur in both guilds.

Species within thermal guilds generally shared thermal distribution edges (Fig. 5a), but the positions of the peak and the degree of skew were more variable (Fig. 5b). A slight negative skew existed when averaged across all species (i.e. warm‐skew, median *T*_*skew*_ = −0.98, IQR = 2.32). *T*_*opt*_ and *T*_*skew*_ were significantly negatively related (Fig. 5b). This was stronger for tropical (β = −0.63 ± 0.02) than temperate guilds (β = −0.40 ± 0.04; *Z* = −5.40, *P *< 0.001), thus the transition from cool‐ to warm‐skew occurs more rapidly along the thermal gradient among tropical species. Contrasting patterns of skew in temperate and tropical species in subtropical regions means that species from different guilds can share a similar *T*_*opt*_, despite having different thermal distribution edges (e.g. Fig. 5c). Model fits were not improved by including taxonomic structure as a proxy for shared evolutionary histories for tropical or temperate species. Results were also robust to inclusion of only ‘high‐confidence’ species (Table S5) and were similar when we used *T*_*min*_ and *T*_*max*_ derived from species’ distribution models or seasonal extremes (Table S4, Figs S11 and S12).

**Figure 6:**
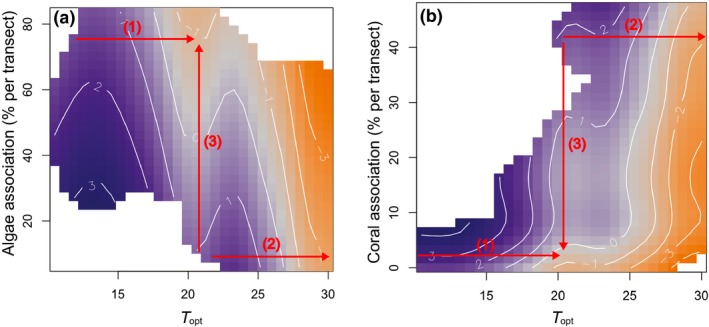
Contour plots showing the response of *T*_*skew*_ to *T*_*opt*_ and species habitat associations: (a) macroalgae, (b) coral. Shading represents cool (blue) to warm (orange) skewed distributions for a given habitat association and thermal optimum. Gradients in *T*_*skew*_ occur not only with increasing *T*_*opt*_ (arrow 1 and 2 – as in Fig. 4b), but also in relation to the local cover of macroalgae and coral at survey sites due to species association with particular habitats (arrow 3). The transition from temperate to tropical fish species is also characterised by a transition from macroalgal to coral habitats, but within this subtropical transition zone *T*_*skew*_ varies sharply with habitat association.

Species' *T*_*opt*_ and habitat associations both independently contributed to the shape of *T*_*skew*_. Within the subtropical transition zone, species that were strongly associated with coral or macroalgae habitats were most strongly skewed, declining in abundance in temperatures where favoured habitats become unavailable (Fig. 6). In contrast, species weakly associated with either habitat type retained abundance across this habitat transition thus having less skewed thermal distributions when optima are located in subtropical temperatures.

## Discussion

Species’ abundances are structured along environmental temperature gradients in a pattern consistent with the abundant‐centre hypothesis. Multiple lines of evidence support this result, first, a peak in maximum abundance occurs in most species. Second, local maximum abundances decline towards each thermal distribution edge at a similar rate. Third, for species that have thermal niche edges available in geographic space (i.e. ranges not constrained by the edges of continents or the warmest seas) almost all show some decline in abundance at thermal distribution edges (97%). Whilst many species display a peak in abundance, truncation of the warm edge for tropical species combined with the fact that there are many more tropical species, leads to a high overall frequency of species displaying warm‐skewed realised abundance distributions (i.e. in our categorical assessment, section *Categorical assessment of thermal‐abundance distribution shape*).

We provide arguably the most robust assessment of patterns of abundance across an environmental gradient to date (but see Santini *et al*. 2018). Our findings contrast previous studies that largely focused on terrestrial or intertidal species (Sagarin & Gaines 2002a; Dallas *et al*. 2017) by finding that species’ abundances frequently display realised niche optima, and that abundance declines at a similar rate towards each niche edge – we interpret this as a signal of a net abundant‐centre pattern. This contrast suggests the effect of climate on ecological performance may be obscured on land by small‐scale processes and factors such as human alterations to habitat availability (i.e. land‐use change) and microclimate variability. Shallow reef fishes could be considered an ideal ‘model’ taxon, given the relatively low thermal heterogeneity and high spatial auto‐correlation of temperature in subtidal marine systems (Steele & Henderson 1994), leading to a reduced capacity to behaviourally thermoregulate (but see Chase *et al*. 2018). Furthermore, terrestrial species which strongly regulate body temperatures – either physiologically (hibernation, endothermy) or behaviourally (burrowing, seeking shade) – may be less likely to show reduced performance at suboptimal temperatures.

Species in each thermal guild have aligned thermal distribution edges (Stuart‐Smith *et al*. 2017). Thus, if all species displayed thermal‐abundance distribution shapes consistent with the abundant‐centre hypothesis, we would observe a local ‘build‐up’ in total community abundance at the centre of each thermal guild (i.e. ~ 17°C and ~ 26°C). However, we found that ecological optima were generally offset relative to each other along the environmental temperature gradient – a pattern we call ‘thermal complementarity’ (Fig. 5). Moving north‐to‐south along coastlines within any given region, there is turnover in the species that are living at their optimal temperature for achieving maximal abundance. Environmental or ecological mechanisms may regulate which species reach their peak abundance along thermal gradients. The mechanisms of this ecological temperature optimisation and segregation require further investigation but could include physiological adaptations to temperature; species interactions partitioning the thermal niche (Attrill & Power 2004; Paterson & Blouin‐Demers 2017); habitat distributions within niche space; or recruitment biases towards particular temperatures or latitudes.

Where thermal guilds turnover rapidly in subtropical regions, the switch in which species are most abundant at any particular site appears to relate to habitat, which transitions from coral to rocky reefs at higher latitudes, but may be dominated by either habitat at subtropical sites. Seasonal temperatures may also prove too extreme for tropical and temperate species living close to their cool and warm thermal limits respectively (Figueira *et al*. 2009). Further work is needed to evaluate the underlying mechanisms of thermal complementarity alongside our correlative approach. Natural experiments in which species’ distributions change (range shifts, invasive species or experimental exploitation), or experimental transplantations (Lee‐Yaw *et al*. 2016) provide opportunities to study the influence of species interactions on ecological performance, and shifts in optima, with altered community structure (Edelist *et al*. 2013).

The different patterns in the skew of the thermal distribution on either side of ~ 23°C are not observed in the critical limits for individual performance from laboratory studies (Fig. S9). Thus, measures of performance for individuals, populations and species may display different responses to temperature, leading to mismatches in predicted responses to temperature change across biological and ecological scales. Always inferring biotic change from laboratory‐based estimates of ‘performance’ could induce systematic biases in predictions of biodiversity change in a warmer world – species never occur in isolation, nor do constant environmental conditions occur in nature. An ensemble of predictive theories and data integration are likely needed from different fields (Sinclair *et al*. 2016).

Beyond subtropical climates, abundance distributions for tropical species were frequently warm‐skewed, but temperate species more frequently displayed cool‐skewed or abundant‐centre patterns. The distributions of the warmest‐affinity tropical species are truncated at their warm thermal distribution edge by the maximum temperatures observed in the oceans. Likewise, the geographic availability of land in southern latitudes may also increase the higher frequency of the cool‐skewed distributions in temperate species. These biogeographic factors aside, studies of metabolic performance report that low‐latitude reef fishes show optimal temperatures near to upper thermal limits, and can rapidly lose function with even a small increase in temperature (Rummer *et al*. 2014). Fundamental thermal niches indicate tropical species across multiple taxa live nearest their upper thermal limit – that is, the ‘hotter is better’ hypothesis – thus the patterns we observe are unlikely to result from a biogeographic boundary effect alone (Deutsch *et al*. 2008; Angilletta *et al*. 2010; Morley *et al*. 2012). We explicitly accounted for the possibility of truncations to thermal‐abundance distributions by only analysing species with absences beyond observed range edges, and through trialling exclusion of species with a *T*_*opt*_ > median *T*_*opt*_ of all tropical species, and the results remained qualitatively unchanged (Tables S4 and S5).

The observational and coarse‐scale nature of our analyses, correlating mean temperature to ecological performance, cannot perfectly exclude other factors influencing ecological performance. For example, we overlook fine‐scale variability in the temperatures experienced by reef fishes which could yield greater understanding of the links between small‐scale temperature variation and ecological performance (e.g. Payne *et al*. 2016). We focus on shallow‐water species, but it is possible that abundance at warm‐range limits is underestimated if species can occupy deeper and cooler reefs (Bates *et al*. 2014). However, shallow and deeper (i.e. mesophotic) reefs are compositionally distinct systems, and so the capacity for deepening at warm‐range limits may be limited (Rocha *et al*. 2018) – such a pattern would still be consistent with the idea that warmer seas reduce species’ maximum abundance potential in shallow‐water ecosystems. In addition, we study a thermal gradient with a correlated transition in dominance from coral to macroalgal cover on reefs (as discussed above; Fig. S10). Although observational analyses are unable to identify temperature as a direct mechanism, our multiple regressions determine that the partial influence of species thermal optima on skew is statistically significant even when correlated habitat associations are considered (Fig. 5, Tables S4 and S5).

The relationship between temperature and maximum abundance suggests at least partial predictability of species‐level maximum abundance response to future temperature changes (Booth *et al*. 2018), and the opportunity to predict changes in maximum abundance across species ranges (Lenoir & Svenning 2013; Martinez‐Gutierrez *et al*. 2018). Such approaches add to estimates of biodiversity change in response to warming that are generally based on changes in occupancy probabilities – changes in abundance are an important component of temperature‐driven biodiversity change, as well as changes in ecosystem function and services (Waldock *et al*. 2018). For example, a change in the number and proportion of individuals within a community comprises a key mechanism whereby biodiversity contributes to ecosystem functions (Winfree *et al*. 2015), and the impact of non‐native and invasive species is tightly linked to abundance (Sofaer *et al*. 2018). In addition, the yield of fisheries depends on the number of individuals in local populations, and the success of marine management is usefully measured by an increase in the number of individuals contributing to community biomass (Edgar *et al*. 2014). Consideration of species’ thermal‐abundance distributions when designating protected areas should enhance biodiversity conservation with climate warming by anticipating, and planning for, species’ abundance increases and declines (rather than just presence) inside protected areas (Fredston‐Hermann *et al*. 2018). These examples implicate the importance of monitoring species’ abundance and quantifying thermal‐abundance distribution shapes to better predict and manage shifting biodiversity in a warming ocean with greater temperature extremes.

## Authorship

All authors contributed to the conceptual development of this manuscript and substantially revised text. Analyses and writing were led by CW.

## Data Availability

Code and data for all analyses are available online (code available at https://github.com/cwaldock1/RLS-ThermalNiche, data available at https://doi.org/10.6084/m9.figshare.7218104).
