# Supplementary material for: The shape of abundance distributions across temperature gradients in reef fishes
Source: Ecol Lett. 2019 Feb 10;22(4):685–96. doi: 10.1111/ele.13222 (PMC6850591; doi:10.1111/ele.13222)
Supplement: Supplementary file 1 [file ELE-22-685-s001.pdf]

Appendix 1. Supplemental methods

Confidence scoring species' thermal distributions

We applied a semi-quantitative confidence scoring system to obtain a test set of 'high confidence' species to ensure our results were robust the limited extent and coverage of observational data. Overall, this filtering by confidence score had little qualitative influence on our results. All species had at least 30 abundance records and were found at sites ranging in > 3°C in any of our analyses. In addition, we assigned a confidence score of 3 and removed points if the criteria in Table 1 were violated. 181 species had a score of 3; 330 a score of 2; 178 a score of 1 and 13 a score of 0. When using T_{skew} values obtained from species' distribution models (i.e., T_{min} and T_{max} were estimated from SDMs rather than observed thermal limits), we applied the additional confidence penalties in Table 2. For these species, 99 had a score of 3; 225 a score of 2; 233 a score of 1 and 115 a score of 0 or less.

Table 1. Confidence scoring criteria applied to ensure results are robust to the following limitations in using observational data.

Criteria	Score deduction	Justification
>10 absences beyond sampling thermal limits	-1	Avoids estimation of realised limits when sampling does not capture range edge
Deviance explained by quantile GAM is > 75th quantile of deviance explained across all species	-1	Subsets to the most predictive abundance models
Standard deviation in estimates of T_{opt}, due to bootstrapping, is > 0.5	-1	Avoids T_{opts} with high uncertainty
T_{opt} does not equal sampling limits	-1	Avoids extrapolation of T_{opt} to beyond sampled temperatures
T_{opt} does not equal T_{max} or T_{min}	-1	Avoids extrapolation of T_{opt} to beyond sampled temperatures

Table 2. Additional confidence scoring criteria applied to SDM model-estimated realised thermal niche edges to ensure results are robust to the following limitations in using model derived niche edges.

Criteria	Score deduction	Justification
T_{max} or T_{min} are 3°C above or below sampling limit	-1	Avoids extrapolation beyond sampling limits
Specificity or sensitivity from species distribution model is < 0.7	-1	Reduces confidence where thermal limit is poorly predicted in SDM
Range of temperatures between T_{min} and T_{max} is not 0.5 – 2x the sampling range	-1	Avoids over or under-prediction of thermal range sizes

Deriving* T_{min} *and* T_{max} *using species' distribution models

To estimate T_{min} and T_{max} we first estimated geographic distributions from an ensemble of 5 species distribution models (SDMs) fitted in the R package 'sdm' independently for each species (Naimi and Araújo 2016). For each species we fitted SDMs using five methods: boosted regression trees, generalized additive models, generalized linear models, supported vector machines, random forests. This combination of approaches is relatively insensitive to correlated covariates (e.g., random forests). With those that create general functional forms (e.g., generalized linear models), we chose approaches best suited to true presence-absence data rather than approaches optimised for pseudo-absences (Elith and others 2006, Guillera-Arroita et al. 2015). We predicted occupancy probabilities from an ensemble of models, which leads to higher accuracy compared to relying on any single underlying model (Araújo and New 2007, Marmion et al. 2009).

We aimed to maximise the predictive capacity of our SDMs by including 15 variables (Table S3) expected to influence species' occupancy rates through effects on: organism physiology (temperature, pH, oxygen), resource availability (primary productivity, nutrient supply), dispersal and establishment capacity of larvae (current velocity and wave strength), exploitation (human population density) and geographic and bathymetric effects (reef area, distance to land). To remove the potential influence of multicollinearity, we reduced the dimensionality of our covariate set for each species using PCA, including only axes with > 10% explained variation. Covariate data were upscaled into ~0.08° grid cells to more closely match the spatial resolutions among the various data sources. Since exploring the influence of each covariate was beyond the aims and scope of this study, we estimated the combined effects of all covariates on species' geographic distributions in an attempt to obtain more accurate estimates of the realised thermal limits than those derived from observational data alone.

The accuracy of each species' SDMs was evaluated by averaging the area under the receiver operator curve (AUC, a standard statistic measuring the performance of binary classifications), specificity (1 - false-presence rate) and sensitivity (1 - false-absence rate), and true-skill (TSS = sensitivity + specificity - 1) statistics across 5-fold cross validations (Fig. S6). Models had good predictive ability with AUC scores of 0.79 ± 0.08, TSS scores of 0.55 ± 0.13. Model ensemble occupancy probabilities were weighted by AUC scores of independent model runs. Whilst AUC statistics can be misleading and confound SDM sensitivity and specificity (Lobo et al. 2008), they are a useful and simple approximation of model predictive accuracy commonly applied to macroecological applications of species distribution models (Visconti et al. 2016, Newbold 2018). We rescaled probabilities of species' occupancy to 0-1 range to ensure probabilities are standardised between species (i.e., some species are naturally more frequent or rare). We next excluded all cells with a value of < 0.1 and estimated the upper and lower thermal edges of species' distributions as the 5th and 95th quantiles of temperature within this relative probability range (estimates of T_{min} and T_{max} were highly correlated regardless of this occupancy threshold, Fig S7).

References

Araújo MB, New M. 2007. Ensemble forecasting of species distributions. Trends in Ecology and Evolution 22:42–47. doi: 10.1016/j.tree.2006.09.010

Elith J, others. 2006. Novel methods improve prediction of species' distributions from occurrence data. Ecography 29:129–151.

Guillera-Arroita G, Lahoz-Monfort JJ, Elith J, et al. 2015. Is my species distribution model fit for purpose? Matching data and models to applications. Global Ecology and Biogeography 24:276–292. doi: 10.1111/geb.12268

Lobo JM, Jiménez-valverde A, Real R. 2008. AUC: A misleading measure of the performance of predictive distribution models. Global Ecology and Biogeography 17:145–151. doi: 10.1111/j.1466-8238.2007.00358.x

Marmion M, Parviainen M, Luoto M, et al. 2009. Evaluation of Consensus Methods in Predictive Species Distribution Modelling. Diversity and Distributions 15:59–69.

Naimi B, Araújo MB. 2016. Sdm: A reproducible and extensible R platform for species distribution modelling. Ecography 39:368–375. doi: 10.1111/ecog.01881

Newbold T. 2018. Future effects of climate and land-use change on terrestrial vertebrate community diversity under different scenarios.

Visconti P, Bakkenes M, Baisero D, et al. 2016. Projecting Global Biodiversity Indicators under Future Development Scenarios. Conservation Letters 9:5–13. doi: 10.1111/conl.12159
