# Supplementary material for: The shape of abundance distributions across temperature gradients in reef fishes
Source: Ecol Lett. 2019 Feb 10;22(4):685–96. doi: 10.1111/ele.13222 (PMC6850591; doi:10.1111/ele.13222)
Supplement: Supplementary file 2 [file ELE-22-685-s002.pdf]

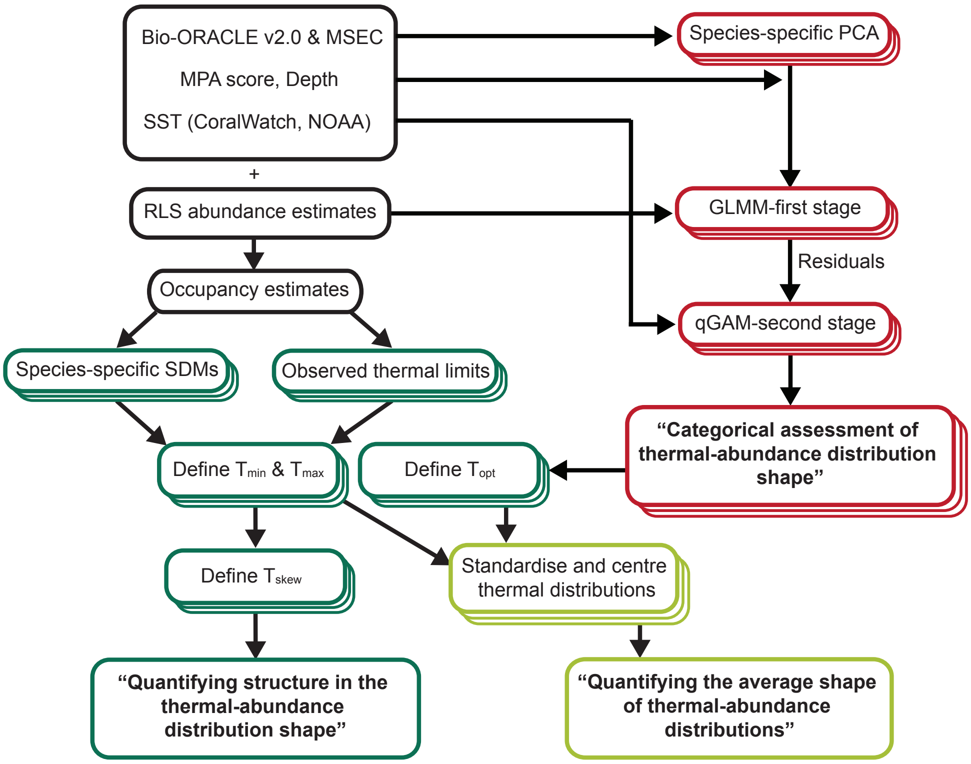

Figure S1. Schematic diagram outlining data and analyses used in the manuscript. Section headings matching methods in main manuscript are in bold. Stacked nodes indicate each species is treated independently.

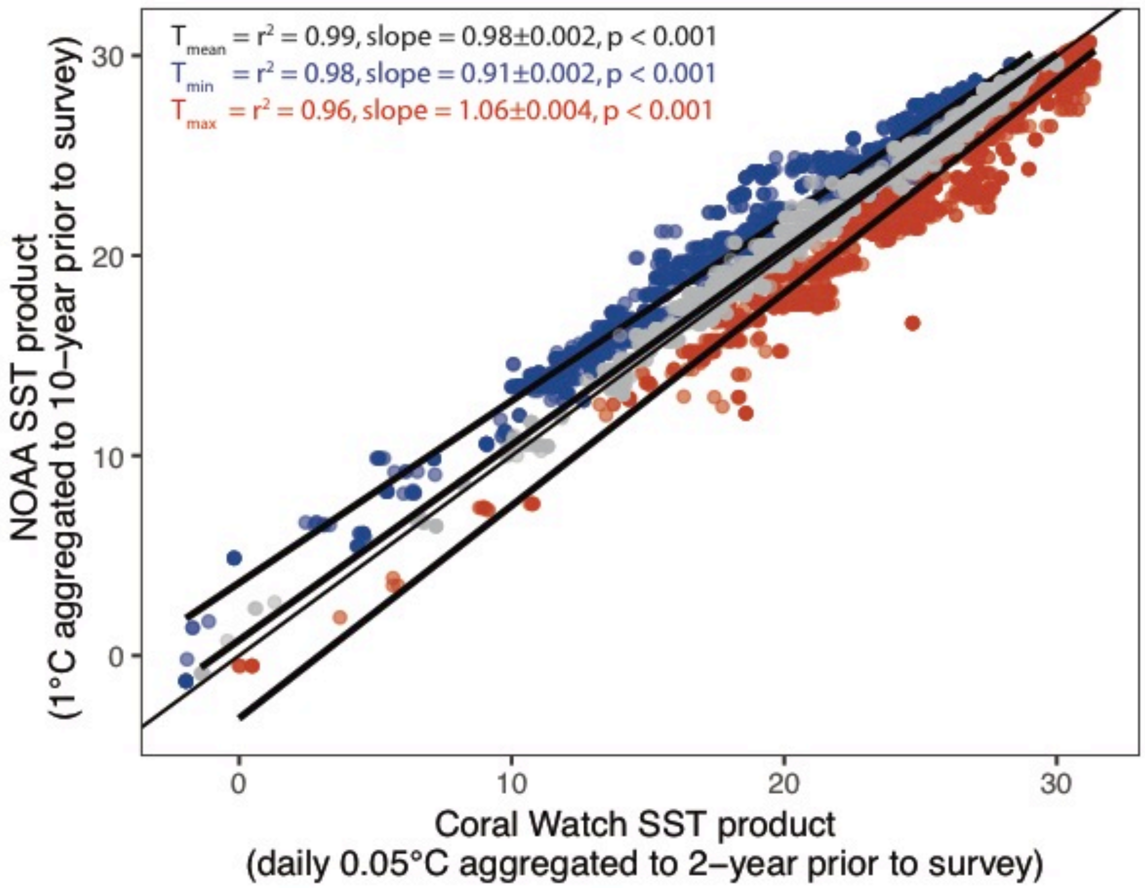

Figure S2. Comparison between satellite sea surface temperatures (SST) for high resolution (Coral Reef Watch) and lower resolution (y-axis, NOAA OI SST V2) data products. Coral Reef Watch data are aggregated to 2 years prior to Reef Life Survey records, and NOAA OI SST V2 data are aggregated to 10 years prior to Reef Life Survey records. Mean SST is almost identical between the SST products at the RLS site scale, displaying an almost 1:1 relationship. There is also extremely high amount of shared variation between data sources when comparing minimum and maximum temperature to NOAA OI SST mean.

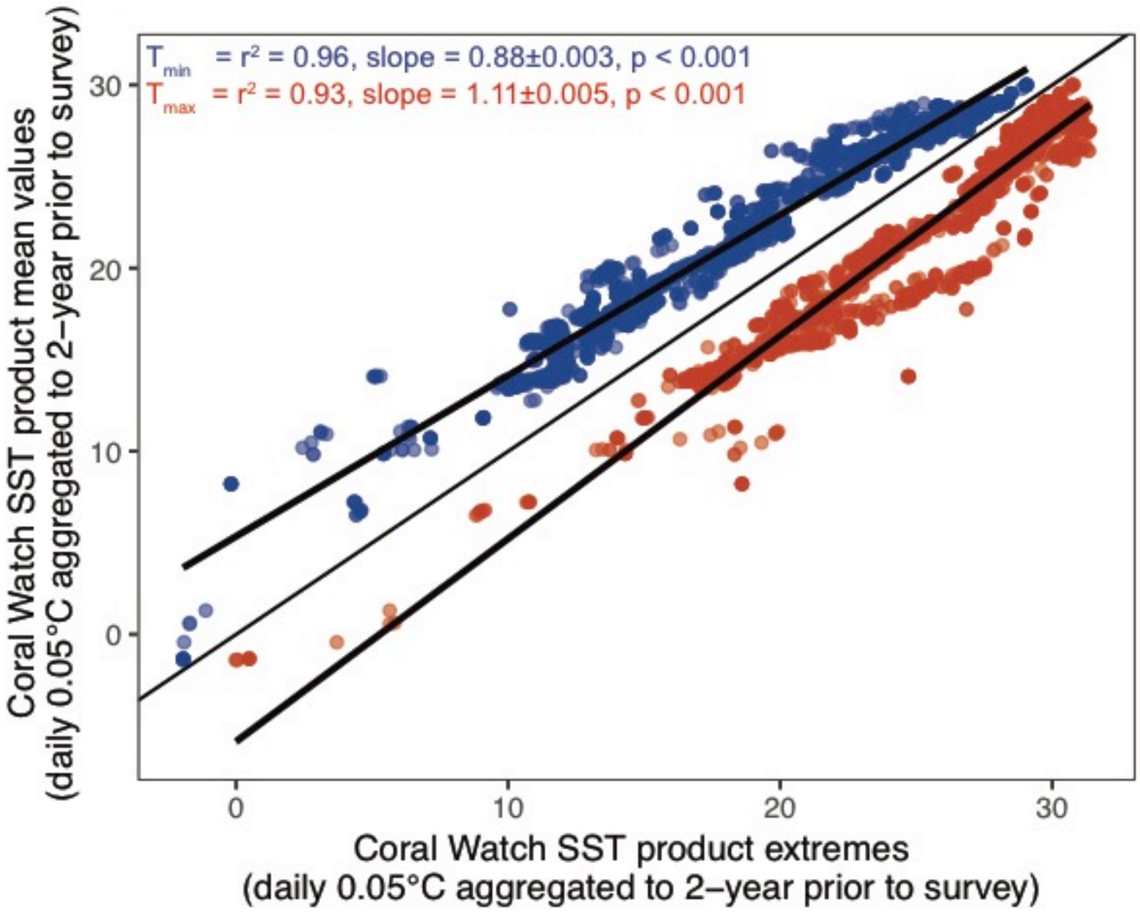

Figure S3. Coral Reef Watch SST product mean values compared to extremes. Very little variation in maximum or minimum temperatures is independent of mean temperature, such that modelling with the mean is adequate to capture the main source of temperature variation at the scales of entire species' geographic ranges (e.g. latitudinal variation in temperatures rather than local to regional features such as ocean upwellings or currents).

Table S1. Covariate data obtain from MSEC and Bio-ORACLE v2.0 for use in modelling abundance. PCAs used rasterized variables only, all variables other than temperature were included in GLMMs prior to fitting quantile model between temperature and local abundance.

Variable name	Unit	Source and reference	Spatial scale	PCA
Depth	m	RLS surveys	Site	
MPA status	NEOLI score	RLS surveys (Edgar et al. 2014)	Site	
Human population density	individuals	MSEC, Yeager et al. (2017)	2.5 arc-minute (~4.6km)	X
Net primary productivity	mg C m^{-2} day^{-1}	MSEC, Yeager et al. (2017)	2.5 arc-minute (~4.6km)	X
Reef area in $15km^2$ (where applicable)	km^2	MSEC, Yeager et al. (2017)	2.5 arc-minute (~4.6km)	X
Current velocity (mean)	M s^{-1}	Bio-ORACLE v2.0, Assis et al. (2018)	5 arc-minute ~(9.2 km)	X
Current velocity (max)	M s^{-1}	Bio-ORACLE v2.0, Assis et al. (2018)	5 arc-minute ~(9.2 km)	X
Dissolved oxygen (min monthly)	mmol m^{-3}	Bio-ORACLE v2.0, Assis et al. (2018)	5 arc-minute ~(9.2 km)	X
Iron	mmol m^{-3}	Bio-ORACLE v2.0, Assis et al. (2018)	5 arc-minute ~(9.2 km)	X
pH	NA	Bio-ORACLE v2.0, Assis et al. (2018)	5 arc-minute ~(9.2 km)	X
Phosphate	mmol m^{-3}	Bio-ORACLE v2.0, Assis et al. (2018)	5 arc-minute ~(9.2 km)	X
Salinity	PSS	Bio-ORACLE v2.0, Assis et al. (2018)	5 arc-minute ~(9.2 km)	X
Silicate	mmol m^{-3}	Bio-ORACLE v2.0, Assis et al. (2018)	5 arc-minute ~(9.2 km)	X
Mean SST	°C	NOAA Coral Reef Watch	2.5 arc-minute (~4.6km)	

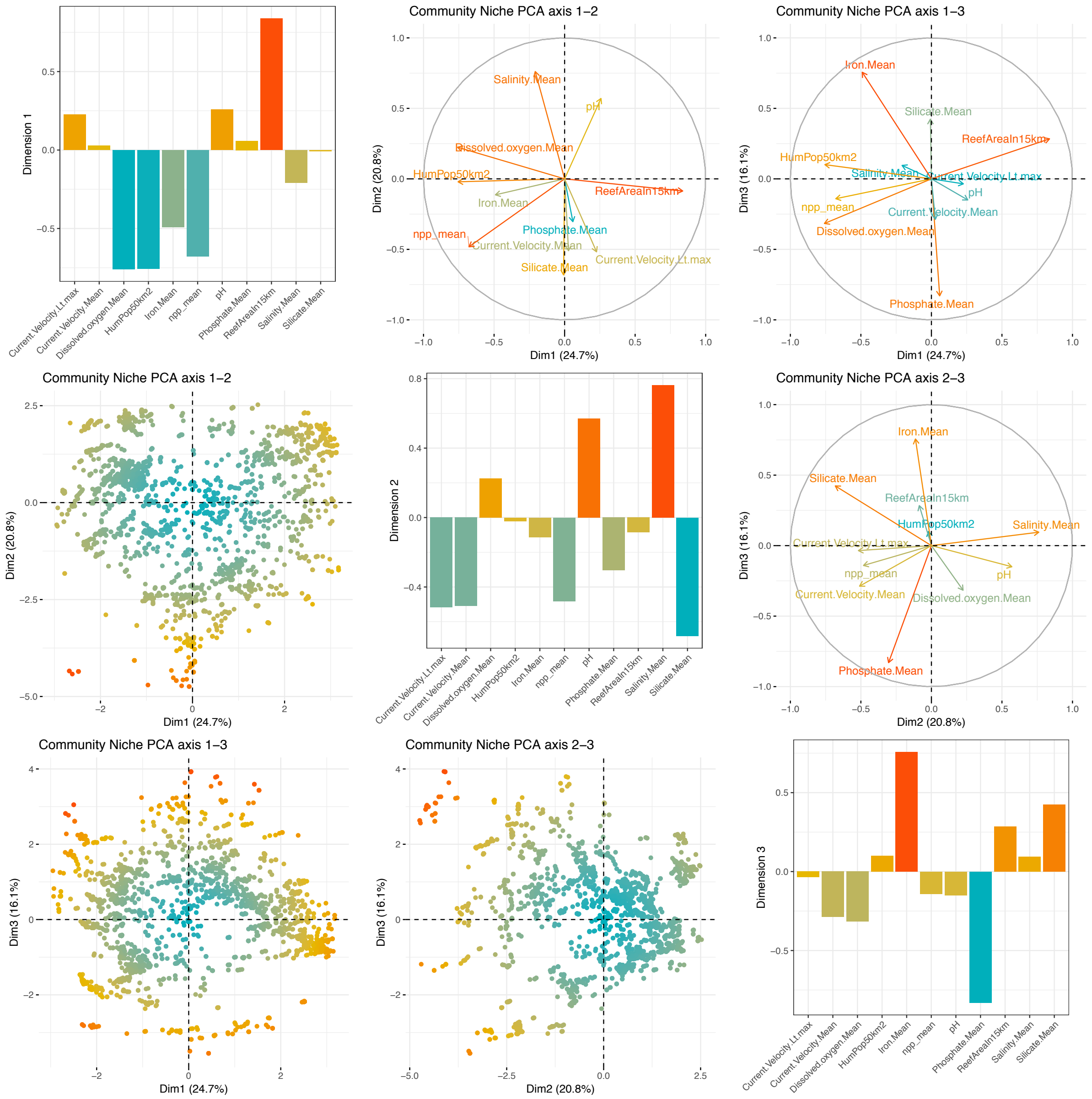

Figure S4. First three axes of the Principal component analysis, which together explain 61% of variation in 11 covariates across all RLS sites together. The major axes of variation are towards low nutrient, isolated reef systems far from human populations (PC1, 25%), towards high salinity, high pH, low silicate systems (PC2, 21%), and towards high iron, low phosphate systems (PC3, 16%).

Table S2. Prior distributions used in JAGS thermal performance curve model, section 2.3.

Parameter	Prior	Limits
T_{opt}	dnorm(0, 0.001)	
σ_{Tmin}	duniform(0, 10)	
σ_{Tmax}	duniform(0, 10)	
c	dnorm(1, 0.001)	0-1

Table S3. Covariate data obtain from MSEC and Bio-ORACLE v2.0 for use in species' distribution models to define T_{min} and T_{min}.

Variable name	Unit	Source and reference	Original spatial scale
Depth	m	GEBCO version 20150318, Weatherall et al. (2015)	30 arc-second (~1km)
Land area in 50km^2	km^2	MSEC, Yeager et al. (2017)	2.5 arc-minute (~4.6km)
Human population density	individuals	MSEC, Yeager et al. (2017)	2.5 arc-minute (~4.6km)
Net primary productivity	mg C m^{-2} day^{-1}	MSEC, Yeager et al. (2017)	2.5 arc-minute (~4.6km)
Reef area in 15km^2 (where applicable)	km^2	MSEC, Yeager et al. (2017)	2.5 arc-minute (~4.6km)
Wave energy flux	kW m-1	MSEC, Yeager et al. (2017)	2.5 arc-minute (~4.6km)
Current velocity	M s^{-1}	Bio-ORACLE v2.0, Assis et al. (2018)	5 arc-minute ~(9.2 km)
Dissolved oxygen (min monthly)	mmol m^{-3}	Bio-ORACLE v2.0, Assis et al. (2018)	5 arc-minute ~(9.2 km)
Nitrate	mmol m^{-3}	Bio-ORACLE v2.0, Assis et al. (2018)	5 arc-minute ~(9.2 km)
Iron	mmol m^{-3}	Bio-ORACLE v2.0, Assis et al. (2018)	5 arc-minute ~(9.2 km)
pH	NA	Bio-ORACLE v2.0, Assis et al. (2018)	5 arc-minute ~(9.2 km)
Phosphate	mmol m^{-3}	Bio-ORACLE v2.0, Assis et al. (2018)	5 arc-minute ~(9.2 km)
Salinity	PSS	Bio-ORACLE v2.0, Assis et al. (2018)	5 arc-minute ~(9.2 km)
Silicate	mmol m^{-3}	Bio-ORACLE v2.0, Assis et al. (2018)	5 arc-minute ~(9.2 km)
Mean SST	°C	Bio-ORACLE v2.0, Assis et al. (2018)	5 arc-minute ~(9.2 km)

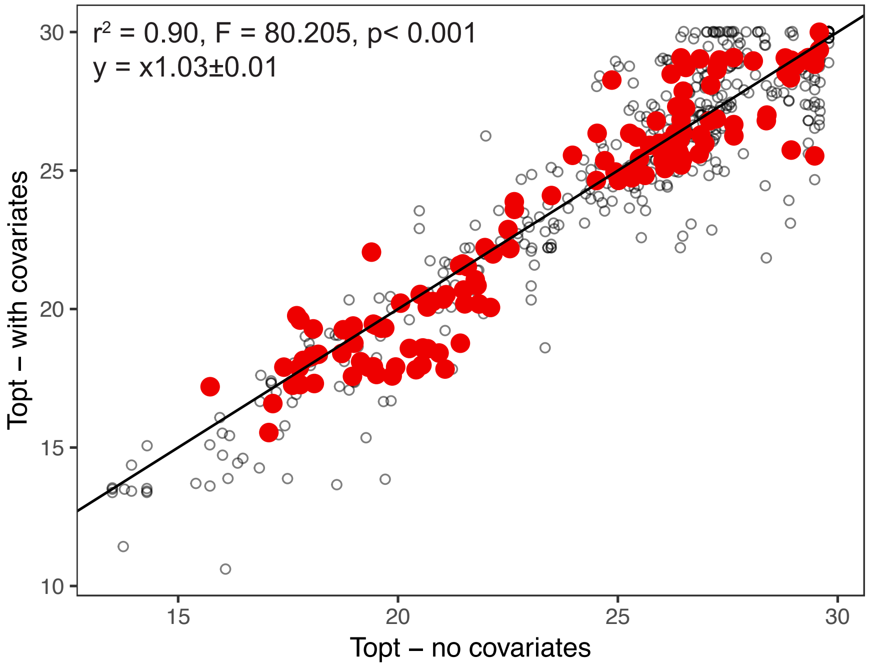

Figure S5. Comparison of T_{opt} values where covariates are included and excluded in model fits (note T_{opt} values without covariates also have coarser 1° x 1° temperature data from preliminary models). Red points indicate high-confidence species.

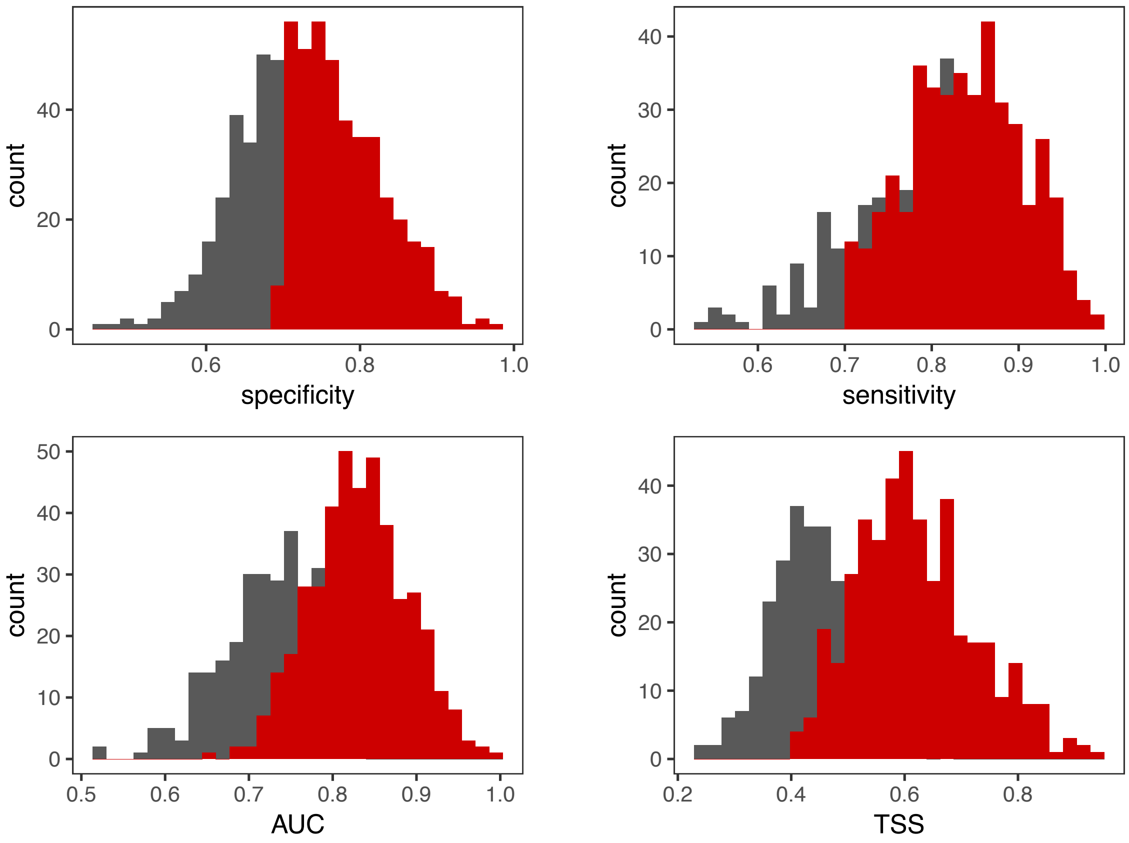

Figure S6. Metrics of species distribution model performance. Stacked histograms showing specificity (presence rate), sensitivity (absence rate), area under the receiver operator curve (AUC) and true-skill statistic (sensitivity + specificity – 1). Red bars represent a 'high-confidence' subset criteria where specificity and sensitivity are > 0.7 and grey bars represent all remaining species.

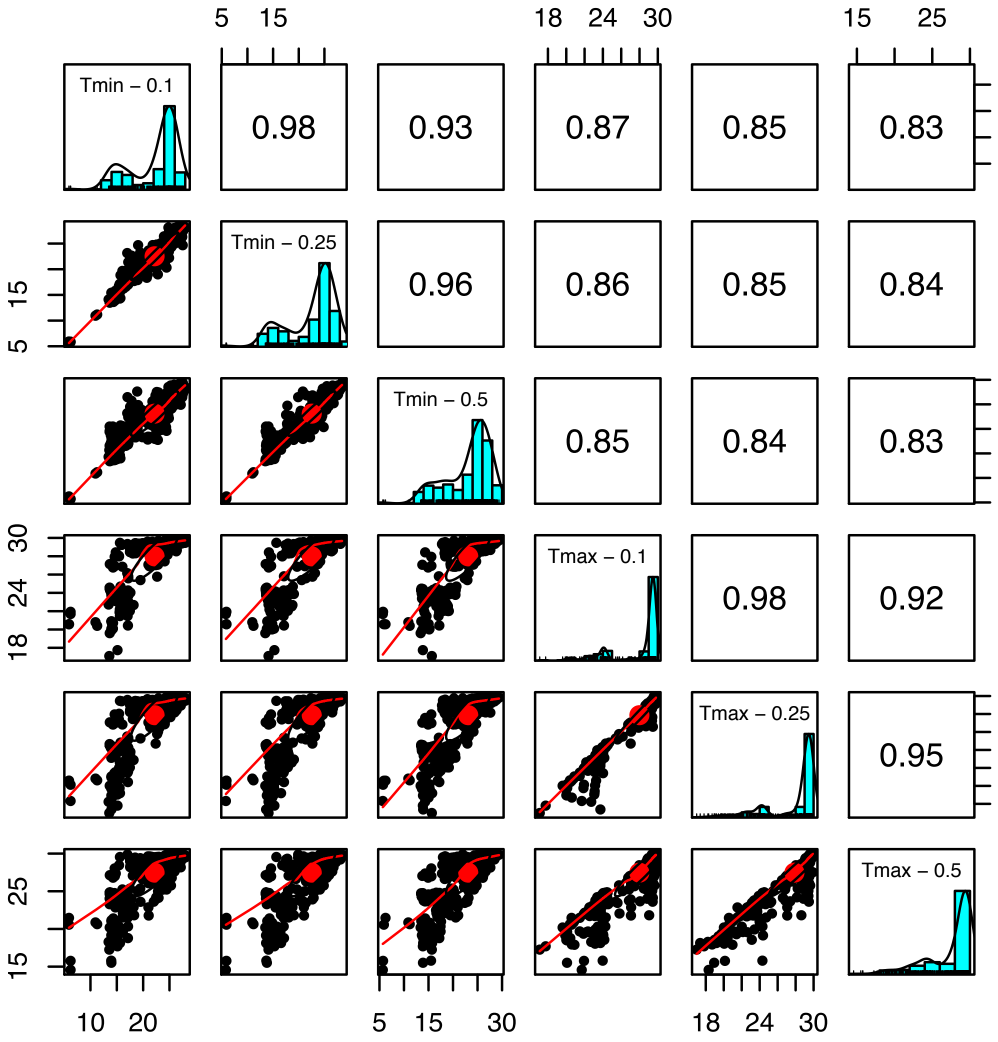

Figure S7. Pairwise correlations between T_{min} and T_{max} estimated based on different threshold values in species distribution models.

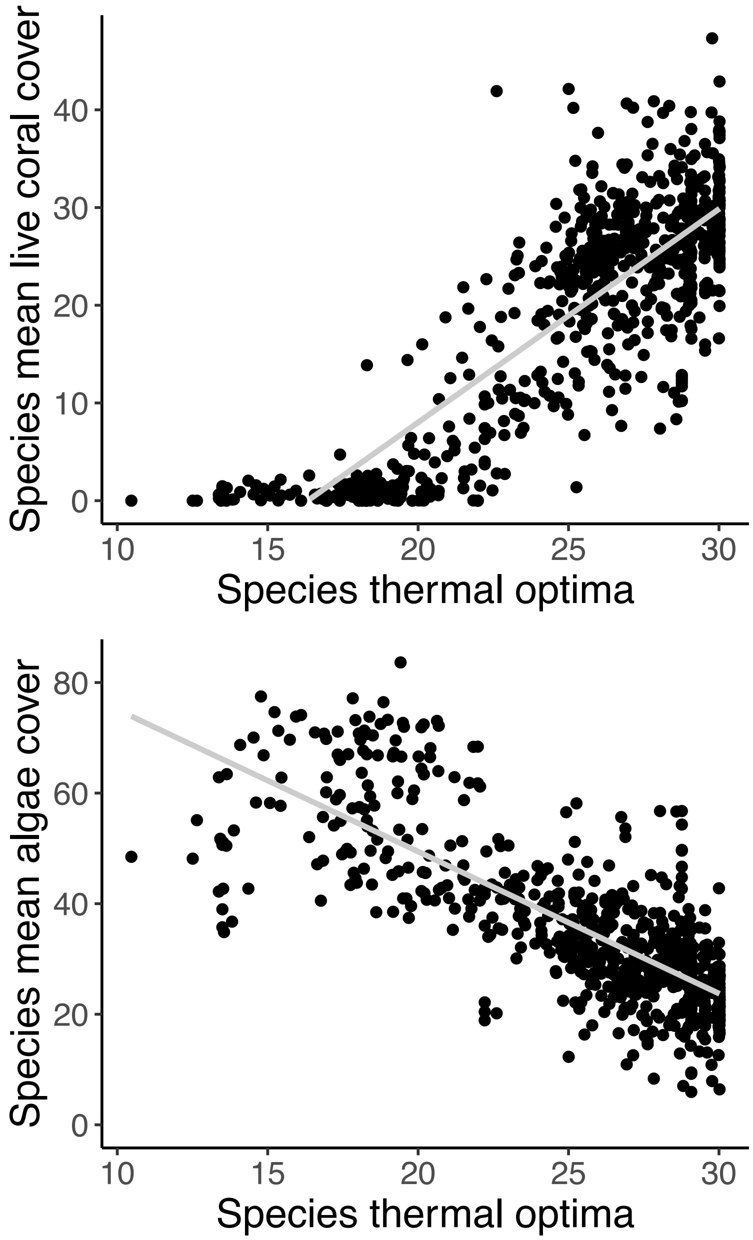

Figure S8. Correlations between T_{opt} and species' coral and algae association.

Table S4. Coefficient summary tables for models predicting T_{skew} including all species. Models are fit to T_{skew} defined in three ways, from observed thermal distribution limits, from observed thermal distribution limits which include seasonality, and from species' distribution models (see methods). Models are fit separately for tropical and temperate species, tropical-2 refers to tropical species with T_{opt} less than the median T_{opt} across all tropical species. Note:*p<0.05; **p<0.01; ***p<0.001.

	T-skew								
	Observed			Seasonal			SDM		
	Tropical	Temperate	Tropical-2	Tropical	Temperate	Tropical-2	Tropical	Temperate	Tropical-2
	(1)	(2)	(3)	(4)	(5)	(6)	(7)	(8)	(9)
T-opt	-0.64***	-0.40***	-0.57***	-0.78***	-0.50***	-0.61***	-0.65***	-0.48***	-0.52*
	(0.02)	(0.04)	(0.05)	(0.02)	(0.04)	(0.05)	(0.08)	(0.10)	(0.24)
Coral association	0.03***	0.11***	0.01	0.05***	0.13***	0.03**	0.07*	0.20	0.15*
	(0.01)	(0.02)	(0.01)	(0.01)	(0.02)	(0.01)	(0.03)	(0.13)	(0.06)
Algae association	-0.01	-0.03***	-0.01	-0.01	-0.03***	-0.004	-0.01	-0.03	-0.06
	(0.01)	(0.01)	(0.01)	(0.01)	(0.01)	(0.01)	(0.03)	(0.02)	(0.05)
Intercept	15.63***	9.54***	14.51***	18.26***	10.90***	14.13***	15.44***	11.36***	12.48
	(0.77)	(0.79)	(1.46)	(0.75)	(0.79)	(1.32)	(2.80)	(1.91)	(6.63)
Observations	524	177	262	524	177	262	74	25	32
Log Likelihood	-668.29	-278.43	-351.84	-657.21	-278.94	-323.40	-113.31	-35.72	-53.11
Akaike Inf. Crit.	1,352.58	572.85	719.68	1,330.41	573.88	662.80	242.62	87.44	122.22
Bayesian Inf. Crit.	1,386.67	598.26	748.22	1,364.50	599.29	691.34	261.05	97.20	133.94

Table S5. Coefficient summary tables for models predicting T_{skew} including only 'high-confidence' species. Models are fit to T_{skew} defined in three ways, from observed thermal distribution limits, from observed thermal distribution limits which include seasonality, and from species' distribution models (see methods). Models are fit separately for tropical and temperate species, tropical-2 refers to tropical species with T_{opt} less than the median T_{opt} and all tropical species. Note:*p<0.05; **p<0.01; ***p<0.001.

	T-skew								
	**Observed**			**Seasonal**			**SDM**		
	Tropical	Temperate	Tropical-2	Tropical	Temperate	Tropical-2	Tropical	Temperate	Tropical-2
	(1)	(2)	(3)	(4)	(5)	(6)	(7)	(8)	(9)
T-opt	-0.67***	-0.43***	-0.61***	-0.83***	-0.43***	-0.74***	-0.65***	-0.41***	-0.63***
	(0.04)	(0.06)	(0.06)	(0.04)	(0.07)	(0.06)	(0.08)	(0.09)	(0.16)
Algae association	0.02	-0.04***	0.04	0.001	-0.05***	0.02	-0.01	-0.04**	-0.03
	(0.01)	(0.01)	(0.02)	(0.01)	(0.01)	(0.02)	(0.03)	(0.01)	(0.05)
Coral association	0.11***		0.14***	0.11***		0.12***	0.07*		0.10
	(0.02)		(0.02)	(0.02)		(0.02)	(0.03)		(0.05)
Intercept	13.55***	10.69***	10.81***	17.98***	10.78***	14.55***	15.44***	10.84***	15.23**
	(1.44)	(1.60)	(2.00)	(1.46)	(1.72)	(2.12)	(2.80)	(1.94)	(5.43)
Observations	130	51	65	130	51	65	74	25	39
Log Likelihood	-147.51	-73.17	-71.50	-151.09	-76.99	-75.20	-113.31	-35.67	-69.24
Akaike Inf. Crit.	311.02	160.34	159.00	318.18	167.99	166.41	242.62	85.34	154.48
Bayesian Inf. Crit.	333.96	173.87	176.40	341.12	181.51	183.80	261.05	93.87	167.79

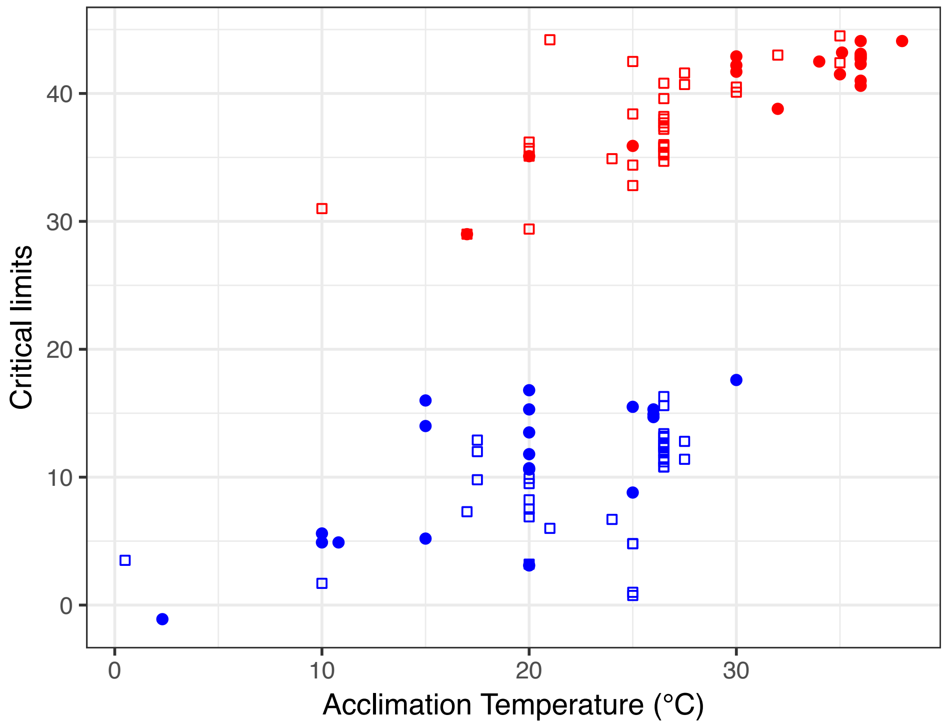

Figure S9. Upper (red) and lower (blue) critical limits for individual performance rates (various traits) of marine fishes in laboratory studies with multiple acclimation temperatures. No obvious signature of thermal guilds is present in either upper or lower critical limits. Data are from Sunday et al. (2011) and GlobTherm (Bennett et al. (2018)), which are shown as filled circles and hollow squares, respectively. These data are the most comprehensive physiological tolerance data available, suggesting that data paucity may always constrain testing thermal guild theory from the physiological literature that has focussed on laboratory studies of individuals over short time-scales.

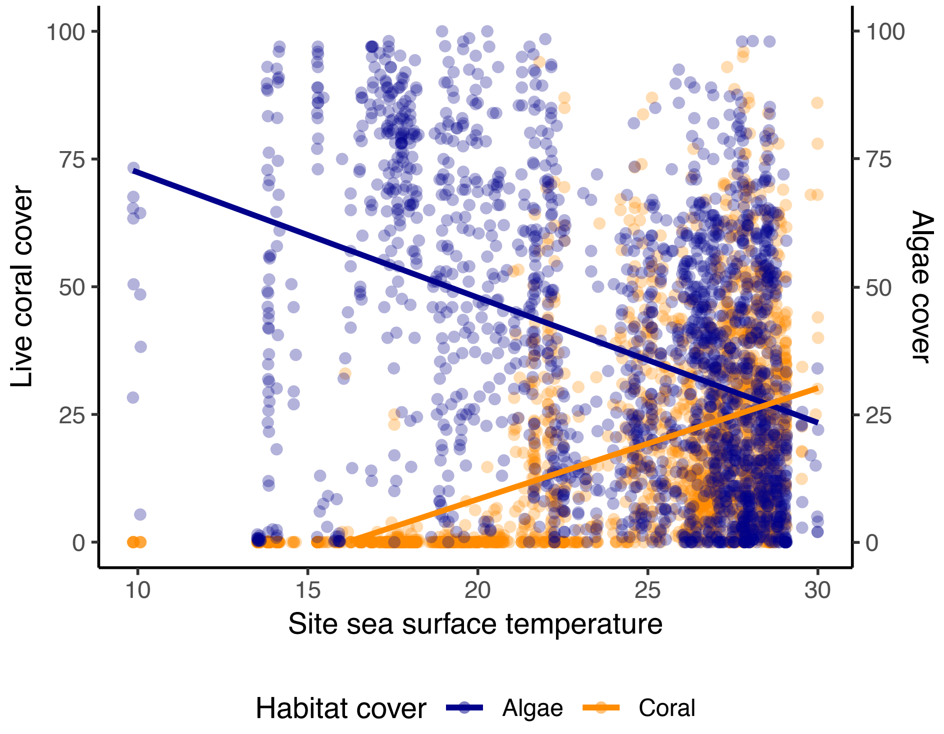

Figure S10. Relationship between algae and coral cover at all sites with site temperature. Note high levels of algae cover even in warmer waters (e.g., >25%).

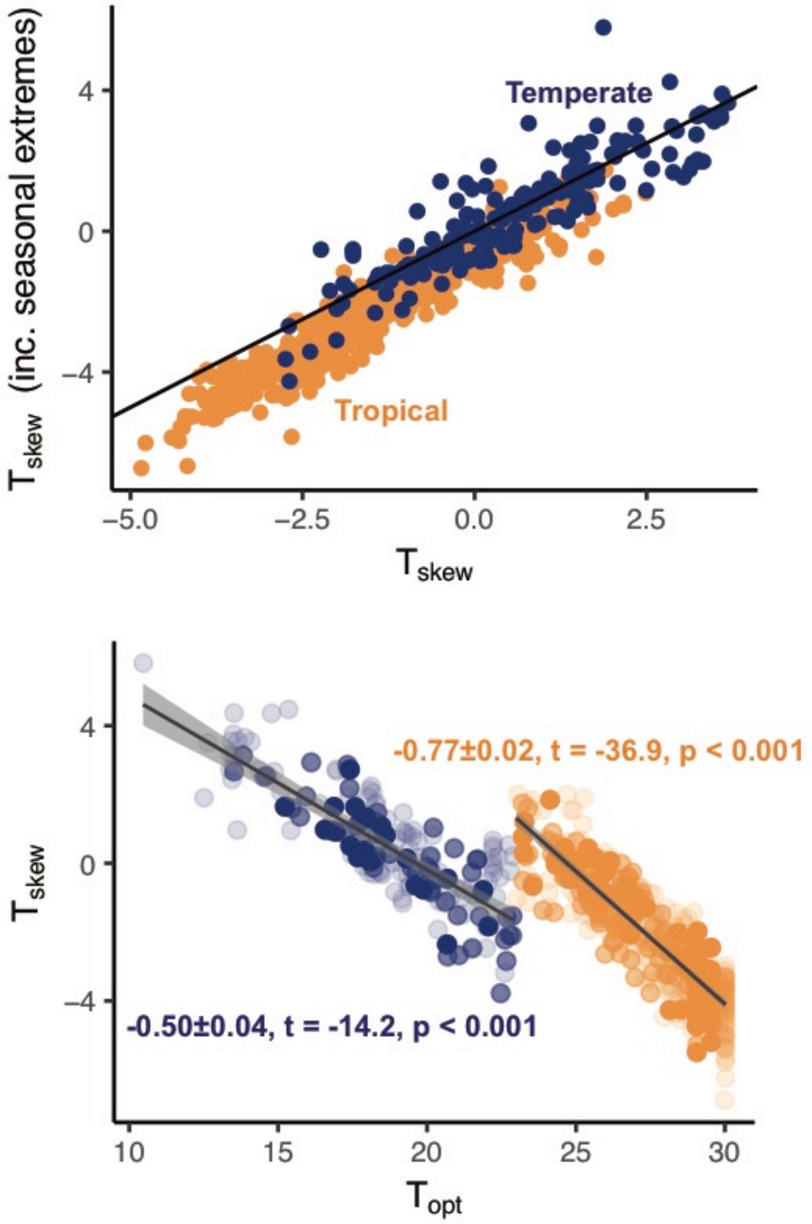

Figure S11. Comparson of main T_{skew} results (i.e., Fig 5) when T_{min} and T_{max} when estimated as the 95th quantiles of minimum and maximum temperature within a 2-year period prior to sampling. Top panel shows T_{skew} excluding seasonal variation compared to T_{skew} including seasonal variation which show a strongly positive slope in both temperate and tropical species. The black line represents a 1:1 relationship. Bottom panel shows T_{skew} vs. T_{opt} as in Fig 5b.

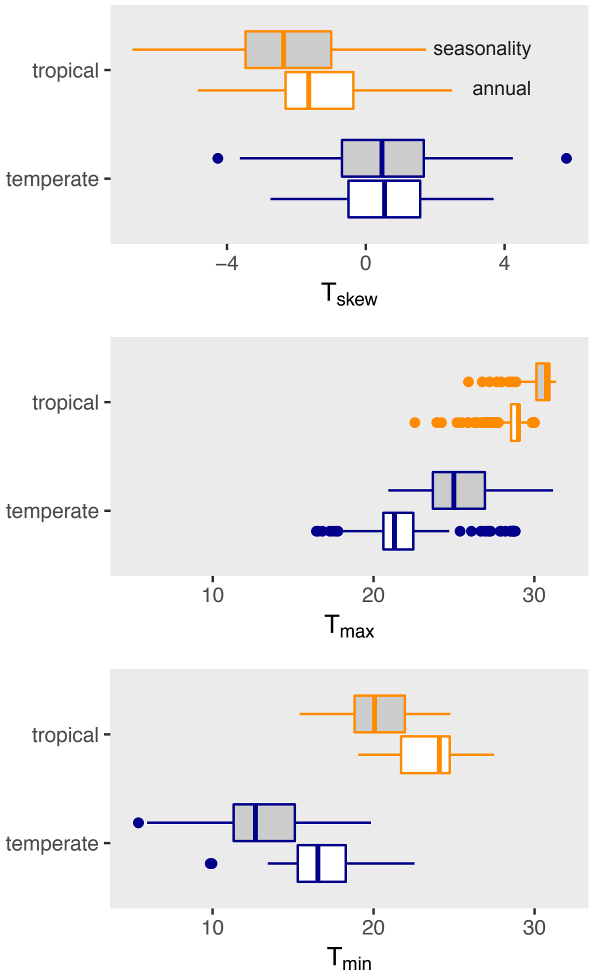

Figure S12. Comparison of T_{skew}, T_{min} and T_{max} when seasonal SST extremes are accounted for (filled gray) in the estimation of upper and lower thermal distribution limits versus when annual mean SST values are used instead (filled white). Tropical (orange) and temperate (blue) species are indicated separately. Including seasonal extremes results in a systematic broadening of thermal niche widths, moreso in tropical species (as in Stuart-Smith et al. 2017). This is balanced between upper and lower limits in each guild, however, and does not strongly influence the skew of species thermal-abundance distributions.
